# Pure Science and Applied Science*

**DOI:** 10.5041/RMMJ.10017

**Published:** 2011-01-31

**Authors:** Robert J. Aumann

**Affiliations:** Nobel Prize Laureate in Economic Sciences, 2005. Professor Emeritus, Center for the Study of Rationality, the Hebrew University, Jerusalem, Israel

It’s a great pleasure and privilege to be here to celebrate 60 years of science in Israel.

The name of my talk is *Pure Science and Applied Science*, and the idea I would like to sell to you today is that there is no such thing as “pure” or “applied” science. In other words, there is such a thing as science, but there is no difference between pure and applied science. Science is one entity and cannot be separated into different categories. In order to back that up, I would like to tell you a little story.

As an undergraduate, I studied mathematics at City College in New York. At that time, what was called Pure Mathematics was in vogue, and the more prominent mathematicians were a little contemptuous of any kind of application. A very famous, prominent mathematician in the first half of the previous century by the name of G. H. Hardy, who was in a branch of mathematics called number theory, said that *the only thing he regretted* was that he unwittingly did some important work in mathematical genetics that eventually turned out to have some application. … Such was the atmosphere in the late ’40s of the previous century and, being a young man and impressionable, I was swept up in this atmosphere.

I also began studying number theory, which deals with prime numbers.

What attracted me, and what attracted Professor Hardy, were four major properties of number theory. One is that the problems in number theory are very natural. The second is that the problems are easy to state. Very often a school-child can understand the statement of the problem in number theory.

Let me give you, as an example, the essence of Fermat’s Theorem. We know that

$$3^{2}+4^{2}=5^{2}$$

But what if, rather than doing it for squares, we try to do it for cubes. Are there three numbers x, y, and z for which x^3^ + y^3^ = z^3^?

People thought about it for a long time and came to the conclusion that the answer is *“No”.*

And how about the 4^th^ power, the 5^th^ power, the 100^th^ power, the 1000^th^ power, anything to the n^th^ power? Is it possible to find x, y, and z, so that x^n^ + y^n^ = z^n^?

This problem was suggested by Mr Fermat 350 years ago, and it remained open for 350 years. People worked on it very hard – for 350 years – until (finally) somebody proved, 15 years ago, that it is impossible, with any n > 2. This is the character of number theory: the problem is natural, easy to state (even the ancient Egyptians knew the first line, namely 3^2^ + 4^2^ = 5^2^), but very difficult to prove. And lastly, and most importantly, it was regarded as absolutely useless. That is what attracted me, so when I got to do my doctorate, I embarked on another subject that appeared to possess all those four attributes.

**Figure 1:**
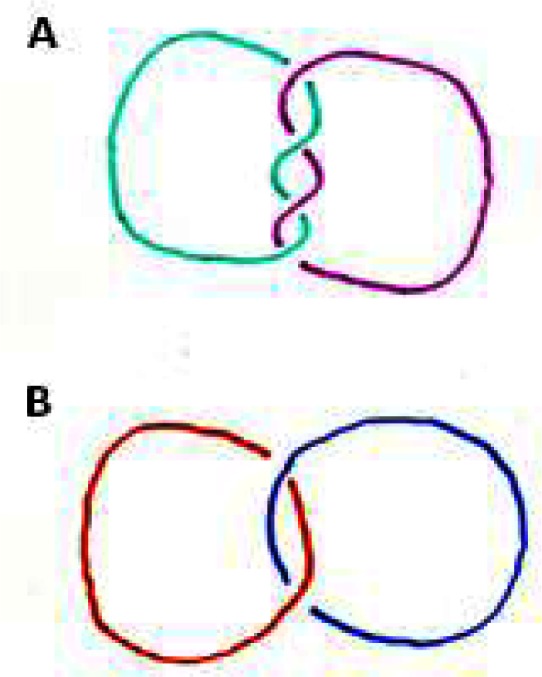
Alternating knots will not come apart.

I did my doctoral thesis in “knot theory”. The problems in knot theory are natural, they are easy to state but difficult to prove, and they are regarded as absolutely useless. Let me tell you what knot theory is about. Figure 1 shows two knots that constitute “alternating knots”, since in these drawings of the knots one line goes over the other one, then under it and over and under again (A); or, one line goes over the other one, then under it (B).

**Figure 2:**
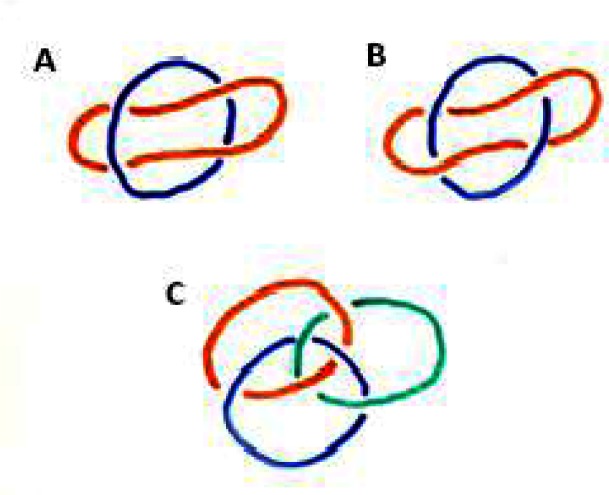
Non-alternating knots (A) may come apart. Alternating knots (B and C) do not come apart.

Figure 2A is an example of a “non-alternating knot”, since one line goes over the other one, again over the same line and then twice beneath it. In Figure 2B one line goes over, under, over, and again under the other line, constituting an “alternating knot”.

In Figure 2C we have line drawings of three knots a red, a green, and a blue. If you follow those three knots along, you will see they alternate. If you look at the one that is non-alternating, you see that you can simply pull it out. In contrast, the ones that are alternating do not seem to come apart.

One of the things I proved in my doctoral thesis is that alternating knots do not come apart, ever. This result is very easy to state; but, like the problems of number theory, it was very difficult to prove. I worked very hard on this problem, and the result had not been known before; and, it is absolutely useless. Who cares! Right? *No!! Wrong!!*

After working on the problem for about two years (1952–54), the basic idea, the breakthrough that was needed to prove this theorem, came to me while standing in the shower in Princeton, New Jersey, one evening in October 1954. Alternating knots do not come apart.

Now, in October of 2004 I sat in my flat (not in the shower) in Jerusalem and the phone rang. It was about 10 p.m. On the line was my grandson, Yacov Rosen, who studied medicine at the university in Beersheva, and he said, “Grandpa, can I pick your brain?” I replied, “Sure, Yacov. What’s up?” He asked, “What are linking numbers?” “Linking numbers, in knot theory?” “Yes,” he said, “in knot theory.” I said, “Why are you interested in linking numbers, why are you interested in knot theory?” He said, “Well, we are studying knot theory in medical school, and the professor talked to us about linking numbers. I didn’t understand what he was saying, and I don’t think he understood it either. So I am asking you: what are linking numbers?”

“Yacov,” I replied, “why are you studying knot theory in medical school? What happened, have they gone crazy in Beersheva? What are they doing?” He said, “Sometimes the DNA in a cell gets knotted up, and, depending on the characteristics of the knot, it can lead to cancer or other problems. So in order to understand what can lead to those problems, we have to understand knot theory.”

I had to sit down, as I was totally bowled over. What I had deliberately chosen to work on 50 years ago *because* it was absolutely useless is now being taught in the second year of medical school in Beersheba, and my grandson is studying it. It was an emotional experience which was really “mind-blowing”. And this is what I want to sell to you: there is no such thing as “pure” and “applied” science. I chose my project because it was so damned “pure” – and now they come and ruin it. They apply it in trying to understand cancer and things like that!!!

I promised Mrs Edith Cresson, the French Prime Minister, to say something about game theory. The original problems of game theory were formulated by John von Neumann, who proved the first important theorem in game theory in 1928 – the Minimax Theory. At the time this was literally a “game”. with no importance whatsoever, on how to solve two-person zero-sum games. Von Neumann was totally unaware of any possible applications of this – until he met Oskar Morgenstern in Princeton 10 years later. Morgenstern, who was an economist, had become aware of this kind of problem from the economics end. The meeting between the two scientists was like two pieces of uranium 239 coming together. The “explosion” that resulted from it created the link between the theory of games and economic behavior. This was published in 1944. The meeting between the mathematician and the economist made the theory of games, initially a “game” of no practical interest – “pure science” – become a cornerstone of modern economic theory and economic practice.

The last item of my talk has to do with Israel and the development of Israel.

Ladies and gentlemen, Israel is the world’s number 2 power in computer technology. The world’s number 1 power is California, and number 2 is Israel. This is due to the school of thought that was founded by Professor Avraham HaLevy Frankel. Professor Frankel, the first chairman of the Mathematics Department at the Hebrew University, was one of the great contributors to “set theory”, axiomatic set theory, in the 1930s through the 1960s. Set theory is a very abstruse branch of mathematics. I would not call it natural and easy to state; it is difficult, and it did seem at the time absolutely useless. Frankel raised a generation of scientists who became interested in what became the foundations of computer science. This is what has made Israel today the number 2 power in computer technology in the world. So again, this very abstruse, this very abstract theory led to the very concrete and important computer technology that we see today.

It works also the other way round. You cannot have pure science that is not in some way rooted in applications. Von Neumann, whom I mentioned earlier, said that things will not work once they become too removed from the real world. You have to think about working in both directions.

There is no such thing as exclusively “pure” or “applied” science, only *good* science. The thing to do is to follow the path upon which your curiosity leads you, and adhere to the principles that govern scientific work.

